# Changes in anticoagulant prescription in Dutch patients with recent-onset atrial fibrillation: observations from the GARFIELD-AF registry

**DOI:** 10.1186/s12959-020-00218-x

**Published:** 2020-03-30

**Authors:** J. Seelig, F. W. A. Verheugt, M. E. W. Hemels, L. Illingworth, A. Lucassen, H. Adriaansen, M. C. M. Bongaerts, M. Pieterse, J. P. R. Herrman, P. Hoogslag, W. Hermans, B. E. Groenemeijer, L. V. A. Boersma, K. Pieper, H. ten Cate, on behalf of the GARFIELD-AF Investigators

**Affiliations:** 1 Department of Cardiology, Rijnstate, Wagnerlaan 55, 6815 AD Arnhem, the Netherlands; 2 Department of Cardiology, Onze Lieve Vrouwe Gasthuis (OLVG), Amsterdam, the Netherlands; 3 Radboud University Medical Centre, Nijmegen, the Netherlands; 4 Thrombosis Research Institute, London, UK; 5 Department of Cardiology, St. Jans Gasthuis, Weert, the Netherlands; 6 Anticoagulation Clinic, Gelre Ziekenhuizen, Apeldoorn-Zutphen, the Netherlands; 7 Anticoagulation Clinic, Ziekenhuis Rivierenland, Tiel, the Netherlands; 8 Stichting Cardiologie Amsterdam, Amsterdam, the Netherlands; 9 Department of Cardiology, Onze Lieve Vrouwe Gasthuis, Amsterdam, the Netherlands; 10 Department of Cardiology, Isala Diaconessenhuis, Meppel, the Netherlands; 11 Department of Cardiology, Elisabeth-TweeSteden Ziekenhuis, Tilburg, the Netherlands; 12 Department of Cardiology, Gelre Ziekenhuizen, Apeldoorn, the Netherlands; 13 Department of Cardiology, St. Antonius Ziekenhuis, Nieuwegein, the Netherlands; 14 Department of Cardiology, Amsterdam UMC, Amsterdam, the Netherlands; 15 Duke Clinical Research Institute, Durham, USA; 16 Department of Internal Medicine, Cardiovascular Research Institute Maastricht, Maastricht, the Netherlands; 17 Anticoagulation Clinic Maastricht, Maastricht, the Netherlands

**Keywords:** Atrial fibrillation, Anticoagulants, Inappropriate prescribing, Guideline adherence

## Abstract

**Background:**

For the improvement of AF care, it is important to gain insight into current anticoagulation prescription practices and guideline adherence. This report focuses on the largest Dutch subset of AF-patients, derived from the GARFIELD-AF registry.

**Methods:**

Across 35 countries worldwide, patients with newly diagnosed ‘non-valvular’ atrial fibrillation (AF) with at least one additional risk factor for stroke were included. Dutch patients were enrolled in five, independent, consecutive cohorts from 2010 until 2016.

**Results:**

In the Netherlands, 1189 AF-patients were enrolled. The prescription of non-vitamin K antagonist oral anticoagulants (NOAC) has increased sharply, and as per 2016, more patients were initiated on NOACs instead of vitamin K antagonists (VKA). In patients with a class I recommendation for anticoagulation, only 7.5% compared to 30.0% globally received no anticoagulation. Reasons for withholding anticoagulation in these patients were unfortunately often unclear.

**Conclusions:**

The data from the GARFIELD-AF registry shows the rapidly changing anticoagulation preference of Dutch physicians in newly diagnosed AF. Adherence to European AF guidelines in terms of anticoagulant regimen would appear to be appropriate. In absence of structured follow up of AF patients on NOAC, the impact of these rapid practice changes in anticoagulation prescription in the Netherlands remains to be established.

## Introduction

In the Netherlands, AF patients on vitamin K antagonist (VKA) therapy are routinely managed by specialized anticoagulation clinics. Back in 2012, a report from the health council of the Netherlands endorsed the careful introduction of NOACs, given the lack of real-world data, absence of specific antidotes, and a substantial risk of non-compliance due to a lack of monitoring [1]. These factors resulted in a slower uptake of a NOAC-based approach in comparison to other countries [2]. However, based on a decision-related Markov model, it was recently calculated that an increase in NOAC prescription in the Netherlands would result in higher quality of life [3, 4]. Moreover, given the increasing real-world data on NOACs versus VKAs, uncertainties about the safety of these drugs have diminished. It is therefore important to monitor anticoagulation prescription trends for AF in the Netherlands, which are currently unknown. This will give insights in how to further improve our AF care.

Moreover, insight in adherence to AF-guidelines could also help to improve AF care. In the Netherlands, it is estimated that the prevalence of AF is around 2.0% in 2020, expected to increase to 3.2% by 2050 [5]. In parallel, in subjects with AF the ischemic stroke rate will rise, primarily due to ageing and an increase in patients with multiple morbidities [5–7]. This increases health-care related costs and reduces quality of life. To minimise these aspects, it is important that AF guidelines are adhered to, as non-adherence is associated with increased ischemic stroke and mortality rates [8, 9].

This report expands on previously published Dutch GARFIELD-AF data, and demonstrates changes in antithrombotic treatment initiation in newly diagnosed AF in the Netherlands [2]. We compare the results with the global GARFIELD-AF cohort, and with recommendations of the most recent European AF-guidelines [10].

## Methods

### Design

GARFIELD-AF was a multicentre, prospective registry of patients with recent onset non-valvular AF from over a 1000 centres in 35 countries worldwide. Globally, the recruitment of patients started in December 2009 and was completed in August 2016. In the Netherlands, patients were included as of November 2010. Patients were enrolled in five independent, consecutive cohorts 1) 2009–2011, 2) 2011–2012, 3) 2013–2014, 4) 2014–2015, and 5) 2015–2016. Data used was from the October 2017 dataset.

### Population

Patients diagnosed with ‘non-valvular’ AF within the previous 6 weeks, aged ≥18 years, and with at least one investigator-determined risk factor for stroke were considered eligible for inclusion. Patients were excluded if; 1) follow-up with a physician was considered unlikely or impossible, 2) there was a potentially reversible, transient cause for AF, or 3) they were enrolled in a controlled clinical trial. For each country, a sufficient number of investigator sites from different care settings were identified.

### Data collection

All data were made anonymous and were imported to a secured, electronic case report form (eCRF), which was designed by Dendrite Clinical Systems Ltd. (Henley-on-Thames, UK). Oversight of operations and data management were done by the Thrombosis Research Institute [TRI] (London, UK), which is the sponsor and coordinating centre. A detailed description of the methods can be found elsewhere [11]. The study is registered at ClinicalTrials.gov (unique identifier: NCT01090362).

At inclusion, patient characteristics such as demographics, medical history, vital signs, and type and dose of antithrombotic therapy were recorded. Amongst others, the components of the CHA_2_DS_2_-VASc stroke risk and HAS-BLED bleeding risk scores were collected [12, 13]. Vascular disease was defined as the combination of a history of acute coronary syndrome with peripheral and/or coronary artery disease. Chronic kidney disease was defined according to the National Kidney Foundation guidelines [14].

### Analysis

Continuous variables are expressed as means with standard deviation, and categorical variables as frequencies with percentages. Data from patients with missing values were not removed from the analyses. Follow-up data was not analysed due to a lack of power. Similarly, no *p*-values were calculated. Data analysis was performed with SAS Enterprise Guide, version 7.1 (SAS Institute Inc., Cary, NC, USA).

## Results

### Population

In the Netherlands, 1189 out of 52,014 patients (2.3%) were enrolled across 16 sites. Across the different Dutch cohorts were 199 (1; 2009–2011), 410 (2; 2011–2012), 357 (3; 2013–2014), 155 (4; 2014–2015), and 161 (5; 2015–2016) AF patients enrolled. In the Netherlands and worldwide, the mean age was 70.7 and 69.7 years, respectively, and 42.4% compared to 44.2% of patients were female. At baseline, hypertension (65.5%), hypercholesterolemia (36.0%), diabetes mellitus (20.0%), and coronary artery disease (18.7%) were the most common comorbidities in the Dutch cohorts. The mean CHA_2_DS_2_-VASc (3.1 vs. 3.2) and HAS-BLED (1.4 vs. 1.4) scores were comparable between the Dutch and overall cohort, respectively. Compared to the worldwide cohort, more patients were enrolled in cardiology departments (90.2% vs. 65.7%) in the Dutch subset. Further baseline characteristics are described in Table 1.

**Table 1:** Baseline characteristics of Dutch and all included patients

	Netherlands (*N* = 1189)	World (*N* = 52,014)
Female sex, n (%)	504 (42.4)	22,987 (44.2)
Age, mean (sd)	70.7 (9.9)	69.7 (11.5)
< 65, n (%)	311 (26.2)	15,693 (30.2)
65–74, n (%)	426 (35.8)	16,948 (32.6)
≥ 75, n (%)	452 (38.0)	19,373 (37.2)
BMI (kg/m^2^), mean (sd)	28.5 (5.3)	27.8 (5.7)
Congestive Heart Failure, n (%)	82 (6.9)	10,397 (20.0)
Hypertension, n (%)	775 (65.5)	39,585 (76.3)
Diabetes Mellitus, n (%)	238 (20.0)	11,540 (22.2)
Stroke/TIA, n (%)	137 (11.5)	5954 (11.4)
PE or DVT, n (%)	22 (1.9)	1356 (2.6)
Coronary artery disease, n (%)	222 (18.7)	11,232 (21.6)
Acute Coronary Syndrome, n (%)	166 (14.0)	4895 (9.5)
Chronic Kidney Disease, n (%)		
None	377 (31.7)	23,919 (46.0)
Stages 1 to 2	629 (52.9)	16,508 (31.7)
Stages 3 to 5	118 (9.9)	5373 (10.3)
History of Bleeding, n (%)	25 (2.1)	1317 (2.5)
Hypercholesterolemia, n (%)	422 (36.0)	20,940 (41.6)
CHA_2_DS_2_-VASc	3.1 (1.5)	3.2 (1.6)
HAS-BLED	1.4 (0.9)	1.4 (0.9)
Care Setting Speciality at Diagnosis, n (%)		
Cardiology	1097 (92.3)	34,165 (65.7)
Other Hospital Departments	30 (2.5)	10,434 (20.1)
Primary Care / General Practice	62 (5.2)	7410 (14.2)

*BMI* Body mass index, *VKA* Vitamin K antagonist, *NOAC* Non-vitamin K antagonist oral anticoagulant, *TIA* Transient ischaemic attack, *PE* Pulmonary embolism, *DVT* Deep venous thrombosis

### Changes in antithrombotic therapy

**Fig. 1:**
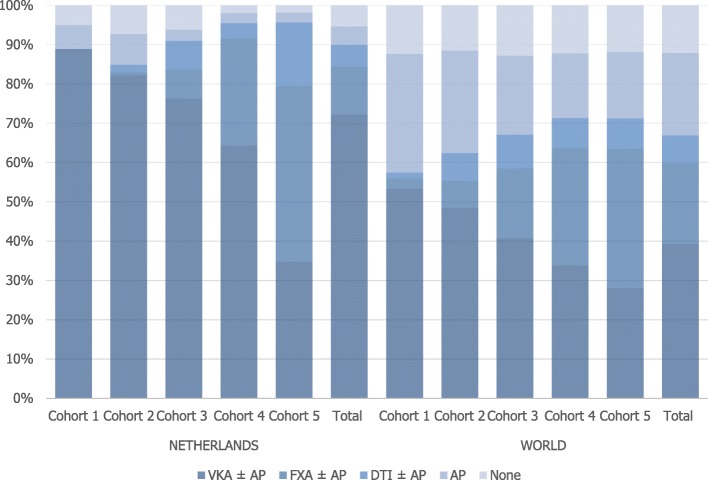
Treatment at diagnosis by cohort (*VKA* Vitamin K Antagonist, *AP* Antiplatelet Drug, *FXa* Factor Xa inhibitor, *DTI* Direct Thrombin Inhibitor)

Of all 35 participating countries, the percentage of patients on oral anticoagulation at AF diagnosis was on average highest in the Netherlands (89.9%). A comparison of anticoagulation treatments (with or without concomitant antiplatelet therapy) between the five different cohorts, demonstrates a rise in the prescription of NOACs from 0.0 to 60.9% over the years (Fig. 1). Conversely, a decrease in VKA prescription from 88.9 to 34.8% was observed. The proportion of patients on antiplatelet monotherapy decreased from 6.1 to 2.5%. The proportion of patients not treated with antithrombotics reduced from 5.1 to 1.9%. In the most recent cohort, the proportion of patients on antiplatelet drug therapy (2.5%) or no antithrombotic therapy (1.9%) were both the lowest of all participating countries.

### Guideline adherence and reasons of not prescribing anticoagulation

**Fig. 2:**
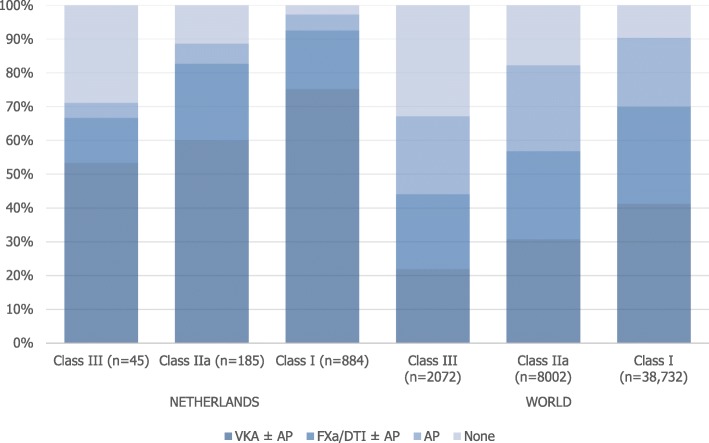
Treatment at diagnosis by Class of Recommendation according to the 2016 ESC AF-guidelines (*VKA* Vitamin K Antagonist, *AP* Antiplatelet Drug, *FXa* Factor Xa inhibitor, *DTI* Direct Thrombin Inhibitor)

Within the Dutch cohorts, 79.4% of patients had a class I recommendation for anticoagulation for stroke prevention in AF (i.e. males CHA_2_DS_2_-VASc ≥2, and females CHA_2_DS_2_-VASc ≥3), according to ESC guidelines [10]. Of these patients, 92.5% were treated with oral anticoagulants, 4.8% with antiplatelet monotherapy, and 2.7% with no antithrombotic therapy (Fig. 2). In patients with a class IIa recommendation for stroke prevention in AF (i.e. males CHA_2_DS_2_-VASc = 1, and females CHA_2_DS_2_-VASc = 2; 16.6% of patients), 82.6% of patients were treated with oral anticoagulants, 6.0% with antiplatelet monotherapy, and 11.4% with no antithrombotic therapy. In patients with no increased stroke risk according to the CHA_2_DS_2_-VASc score (i.e. males CHA_2_DS_2_-VASc = 0, and females CHA_2_DS_2_-VASc = 1; 4.0% of patients), 66.7% were treated with oral anticoagulants, 4.4% with antiplatelet monotherapy, and 28.9% with no antithrombotic therapy.

Unfortunately, in the Netherlands and worldwide, reasons for not prescribing anticoagulants in males with CHA_2_DS_2_-VASc ≥2, and females with CHA_2_DS_2_-VASc ≥3 were often recorded as ‘unknown’ (28.8% versus 39.4%) or ‘other’ (40.9 versus 22.4%). Excluding these options, the most frequently reported reasons in the Netherlands were ‘low stroke risk’ (12.1%) and ‘bleeding risk’ (7.6%) (Table 2). In the worldwide cohort, excluding Dutch patients, the main reasons for not prescribing anticoagulants were ‘patient refusal’ (7.8%), ‘bleeding risk’ (7.2%), ‘low risk of stroke’ (5.8%) and ‘already taking antiplatelet drugs for other medical condition’ (5.4%).

**Table 2:** Main reasons anticoagulant not used in males with CHA_2_DS_2_-VASc ≥2, and females with CHA_2_DS_2_-VASc ≥3

	Netherlands (*N* = 66) n (%)	World (*N* = 11,630) n (%)
Alcohol abuse	0 (0.0)	48 (0.4)
Already taking AP for other medical condition	3 (4.5)	628 (5.4)
Patient refusal	1 (1.5)	911 (7.8)
Previous bleeding event	2 (3.0)	211 (1.8)
Taking medication contraindicated or cautioned for use with OAC	1 (1.5)	78 (0.7)
Other	12 (18.2)	1682 (14.5)
Unknown	19 (28.8)	4588 (39.4)
Physician’s choice	28 (42.4)	3484 (30.0)
Bleeding risk	5 (7.6)	836 (7.2)
Concern over patient compliance	0 (0.0)	412 (3.5)
Guideline recommendation	0 (0.0)	237 (2.0)
Fall risk	0 (0.0)	401 (3.4)
Low risk of stroke	8 (12.1)	677 (5.8)
Other	15 (22.7)	921 (7.9)

*AP* Antiplatelet drug, *OAC* Oral anticoagulation

## Discussion

GARFIELD-AF was the largest, worldwide, prospective registry of newly diagnosed AF patients. In the Netherlands, 1189 patients were enrolled, making it the largest Dutch AF-cohort available to date. This manuscript provides a unique insight in the rapid changes in anticoagulation management of novel AF, which had not been described since the introduction of the NOACs in the Netherlands. The comparison between NOAC uptake rates in the Netherlands vs other countries is important, as this could have influenced the quality of Dutch AF care. Future studies will have to analyze how these differences have impacted the safety and efficacy of AF care. Moreover, this is the first report describing nationwide adherence to AF-guidelines in the Netherlands and explores reasons for withholding oral anticoagulation in AF, which gives insight in how to further improve our AF care. Also, this country-specific evaluation may also be of help in improving care when comparisons are made with anticoagulant management in other countries.

In the Netherlands, there was initially a slow shift to more NOAC prescription, compared to the rest of the world. However, as of 2014–2015, the anticoagulation landscape has changed rapidly, resulting in more newly diagnosed AF patients treated with NOACs than VKA as of 2016. Our findings were comparable to a recent analysis of anticoagulant pharmaceutical dispensing data of naïve oral anticoagulation starters for any indication in the Netherlands [15]. A possible explanation for this initial slow shift could be that there is a well-organized system of specialized anticoagulation clinics in the Netherlands. In these clinics, the monitoring of compliance and complications of VKA treatment through regularly scheduled follow-up checks is aimed at minimising risks accompanying VKA treatment. Although NOACs have been repeatedly shown to be at least as effective and safe as VKAs in both randomized controlled trials and real-world data, a lack of monitoring could have contributed to a hesitation to shift to a more NOAC based approach. This is not unreasonable, as without a regular check of factors such as renal function, weight or age, patients are often (± 10% in two recent Dutch AF-studies), treated with a too high or too low NOAC dose [16, 17]. Moreover, early discontinuation of (N)OAC treatment can be as high as 50% at 6 months in certain patient groups [18, 19]. Frequently mentioned reasons for early discontinuation are (minor) bleeding, other anticoagulant-related side-effects, and a lack of the perceived need for anticoagulation [20, 21]. Therefore, international guidelines recommend structured follow up of patients on NOACs (ESC) including assessment of adherence to medication, complications, interactions and regular (at least annual, but more often on indication) check on renal and liver functions [22]. For the Netherlands, much of this burden will come down on the shoulders of prescribers (mainly cardiologists) and for the long term on general practitioners. It is imperative that, based on national guidance documents such as the “Landelijke Standaard Ketenzorg Antistolling” (LSKA) 2.0 and the updated “Landelijke Transmurale Afspraak antistolling” (manuscript in preparation), the chronic care for patients on NOACs becomes well organized [23].

In GARFIELD-AF, the Netherlands had the highest proportion of patients on oral anticoagulation at diagnosis (89.9%). In the most recent cohort, Dutch patients had the lowest proportions of antiplatelet monotherapy (2.5%) or no antithrombotic therapy (1.9%). For patients with a class I recommendation for anticoagulation, 7.5% of patients were undertreated according to the ESC guidelines [10]. Compared to the worldwide cohort (30.0%), this proportion is relatively low. In patients with a class III recommendation for anticoagulation (i.e. CHA_2_DS_2_-VASc 0 in males, CHA_2_DS_2_-VASc 1 in females), the proportion of patients on anticoagulation is high (66.6%) [10]. Although there is no chronic indication for anticoagulation in these patients, the guideline recommends at least 3 weeks of pre-treatment with oral anticoagulation in late cardioversions [10]. The ACWAS trial showed that in patients with recent-onset (< 36 h) AF, a delayed cardioversion strategy led to spontaneous conversion within 48 h in 69% of patients [24]. In a post-hoc analysis of the ACUTE trial, nearly 50% of patients with pre-existing AF of ≤1 week had a spontaneous cardioversion [25]. It is likely that patients with recent-onset, newly diagnosed AF without risk factors for stroke are often ‘overtreated’ with anticoagulation, given the high rate of spontaneous conversion. It is therefore worth researching if there are possibilities to safely limit the prescription of anticoagulants in these patients.

Although the proportion of undertreated patients in the Netherlands was relatively low, there is still room for improvement. In GARFIELD-AF, main reasons for not prescribing anticoagulants in patients with a class I recommendation for anticoagulation for stroke prevention in AF were often not clear. In patients with a clear recorded reason for withholding anticoagulation, a ‘low risk of stroke’ (12.1%) and ‘bleeding risk’ (7.6%) were the most common reasons in the Dutch cohort. Depicting patients with 2 or more non-sex related stroke risk factors as having a ‘low risk of stroke’ is contradictory, and the precise reasoning behind it is unknown. It would be valuable to gather more information on reasons for withholding anticoagulation, and to evaluate if withholding anticoagulation in these groups is a safe approach.

This study has several limitations. As described before, the high proportion of patients included in Dutch cardiology departments limits the external validity of this study to nationwide clinical practice. Moreover, the number of patients was too low, and the mean follow-up was too short, to relate major adverse events to CHA_2_DS_2_-VASc scores or changes in anticoagulant treatment practices. Moreover, reasons for not prescribing anticoagulants were extracted from the medical records and were not confirmed by the prescribing physician, and a large proportion of reasons could not be recorded and were classified as ‘other’. Further research without these limitations is necessary. DUTCH-AF (Dutch trial register number: NL7464) is a largescale registration of newly diagnosed AF-patients in the Netherlands, which does not have these limitations and could provide further answers [26].

## Conclusion

The data from the GARFIELD-AF registry shows the rapidly changing anticoagulation preference of Dutch physicians in newly diagnosed AF. Adherence to European AF guidelines in terms of anticoagulant regimen would appear to be appropriate. In absence of structured follow up of AF patients on NOAC, the impact of these rapid practice changes in anticoagulation prescription in the Netherlands and in relation to other countries remains to be established.

## Data Availability

All data generated or analysed during this study are included in this published article.

## References

[1] New anticoagulants - a well-dosed introduction: health council of the Netherlands. 2012. [27-Jan-2020]. Available from: https://www.healthcouncil.nl/documents/advisory-reports/2012/05/15/new-anticoagulants-a-well-dosed-introduction.

[2] Ten Cate V, Ten Cate H, Verheugt FW (2016). The Global Anticoagulant Registry in the FIELD-Atrial Fibrillation (GARFIELD-AF): exploring the changes in anticoagulant practice in patients with non-valvular atrial fibrillation in the Netherlands. Neth Hear J.

[3] Verhoef TI, Redekop WK, Hasrat F, de Boer A, Maitland-van der Zee AH (2014). Cost effectiveness of new oral anticoagulants for stroke prevention in patients with atrial fibrillation in two different European healthcare settings. Am J Cardiovasc Drugs.

[4] de Jong LA, Koops M, Gout-Zwart JJ (2018). Trends in direct oral anticoagulant (DOAC) use: health benefits and patient preference. Neth J Med.

[5] Krijthe BP, Kunst A, Benjamin EJ (2013). Projections on the number of individuals with atrial fibrillation in the European Union, from 2000 to 2060. Eur Heart J.

[6] Son MK, Lim NK, Park HY (2018). Trend of prevalence of atrial fibrillation and use of oral anticoagulation therapy in patients with atrial fibrillation in South Korea (2002-2013). J Epidemiol.

[7] Proietti M, Laroche C, Nieuwlaat R (2018). Increased burden of comorbidities and risk of cardiovascular death in atrial fibrillation patients in Europe over ten years: a comparison between EORP-AF pilot and EHS-AF registries. Eur J Intern Med.

[8] Kirchhof P, Ammentorp B, Darius H (2014). Management of atrial fibrillation in seven European countries after the publication of the 2010 ESC guidelines on atrial fibrillation: primary results of the PREvention oF thromboemolic events--European Registry in Atrial Fibrillation (PREFER in AF). Europace..

[9] Gorin L, Fauchier L, Nonin E (2011). Prognosis and guideline-adherent antithrombotic treatment in patients with atrial fibrillation and atrial flutter: implications of undertreatment and overtreatment in real-life clinical practice; the Loire Valley atrial fibrillation project. Chest..

[10] Kirchhof P, Benussi S, Kotecha D (2016). 2016 ESC guidelines for the management of atrial fibrillation developed in collaboration with EACTS. Eur Heart J.

[11] Kakkar AK, Mueller I, Bassand JP (2012). International longitudinal registry of patients with atrial fibrillation at risk of stroke: Global Anticoagulant Registry in the FIELD (GARFIELD). Am Heart J.

[12] Lip GY, Nieuwlaat R, Pisters R, Lane DA, Crijns HJ (2010). Refining clinical risk stratification for predicting stroke and thromboembolism in atrial fibrillation using a novel risk factor-based approach: the euro heart survey on atrial fibrillation. Chest..

[13] Pisters R, Lane DA, Nieuwlaat R (2010). A novel user-friendly score (HAS-BLED) to assess 1-year risk of major bleeding in patients with atrial fibrillation: the euro heart survey. Chest..

[14] International Society of Nephrology. Chapter 2: definition, identification, and prediction of CKD progression. Kidney Int Suppl. 2013;3(1):63–72. 10.1038/kisup.2012.65 PMC4089637 25018976

[15] van den Heuvel JM, Hovels AM, Buller HR (2018). NOACs replace VKA as preferred oral anticoagulant among new patients: a drug utilization study in 560 pharmacies in the Netherlands. Thromb J.

[16] Jacobs MS, van Hulst M, Campmans Z, Tieleman RG (2019). Inappropriate non-vitamin K antagonist oral anticoagulants prescriptions: be cautious with dose reductions. Neth Hear J.

[17] Pisters R, van Vugt SPG, Brouwer MA (2017). Real-life use of rivaroxaban in the Netherlands: data from the Xarelto for prevention of stroke in patients with atrial fibrillation (XANTUS) registry. Neth Hear J.

[18] Harper P, Pollock D, Stephens M (2018). Dabigatran persistence and adherence in New Zealand: a nationwide retrospective observational study. BMJ Open.

[19] Beyer-Westendorf J, Ehlken B, Evers T (2016). Real-world persistence and adherence to oral anticoagulation for stroke risk reduction in patients with atrial fibrillation. Europace..

[20] Lip GYH, Pan X, Kamble S (2018). Discontinuation risk comparison among ‘real-world’ newly anticoagulated atrial fibrillation patients: Apixaban, warfarin, dabigatran, or rivaroxaban. PLoS One.

[21] McHorney CA, Spain CV (2011). Frequency of and reasons for medication non-fulfillment and non-persistence among American adults with chronic disease in 2008. Health Expect.

[22] Steffel J, Verhamme P, Potpara TS (2018). The 2018 European heart rhythm association practical guide on the use of non-vitamin K antagonist oral anticoagulants in patients with atrial fibrillation. Eur Heart J.

[23] Landelijke Standaard Keten Antistolling 2.0 (LSKA): KNMP. Available from: https://www.knmp.nl/patientenzorg/samenwerking/landelijke-standaard-keten-antistolling-2-0-lska. Accessed 27 Jan 2020.

[24] Pluymaekers N, Dudink E, Luermans J (2019). Early or delayed cardioversion in recent-onset atrial fibrillation. N Engl J Med.

[25] Tejan-Sie SA, Murray RD, Black IW (2003). Spontaneous conversion of patients with atrial fibrillation scheduled for electrical cardioversion: an ACUTE trial ancillary study. J Am Coll Cardiol.

[26] DUTCH-AF Registry (2018). Prospective evaluation of dosing and adherence of anticoagulant treatment and the risk for bleeding in atrial fibrillation: Nederlands Trial Register.

